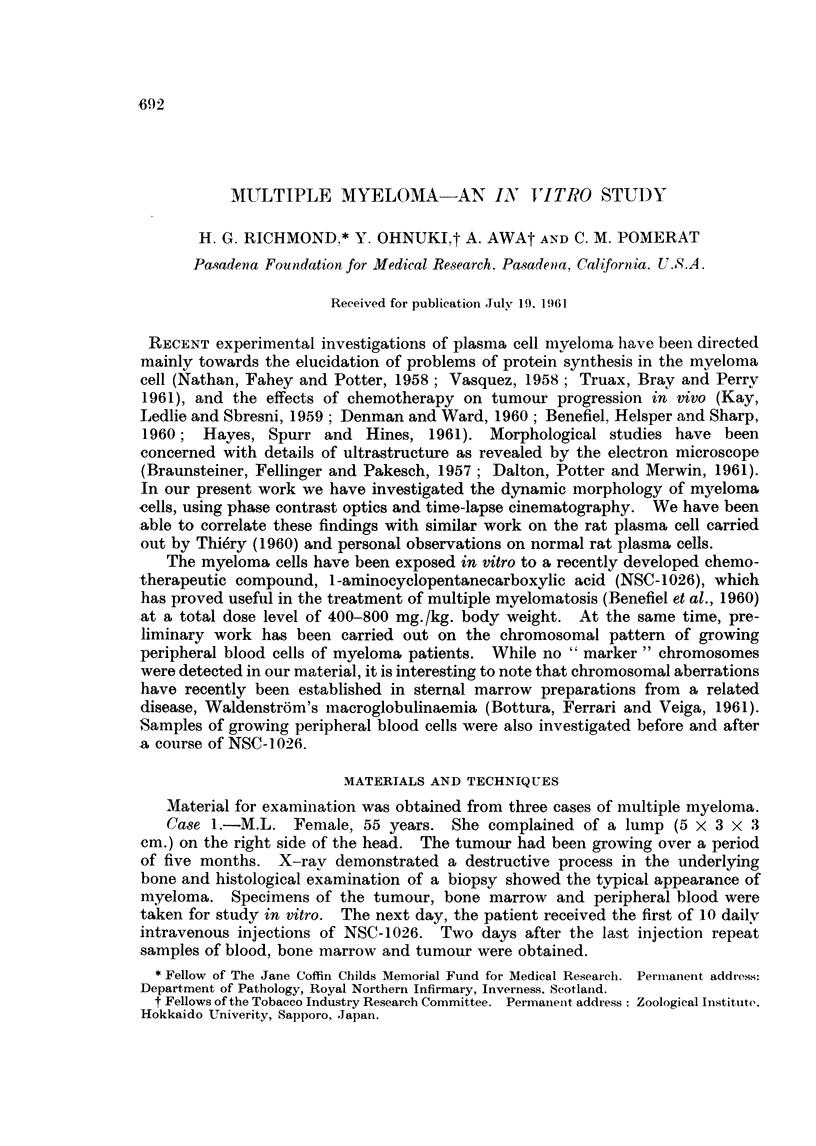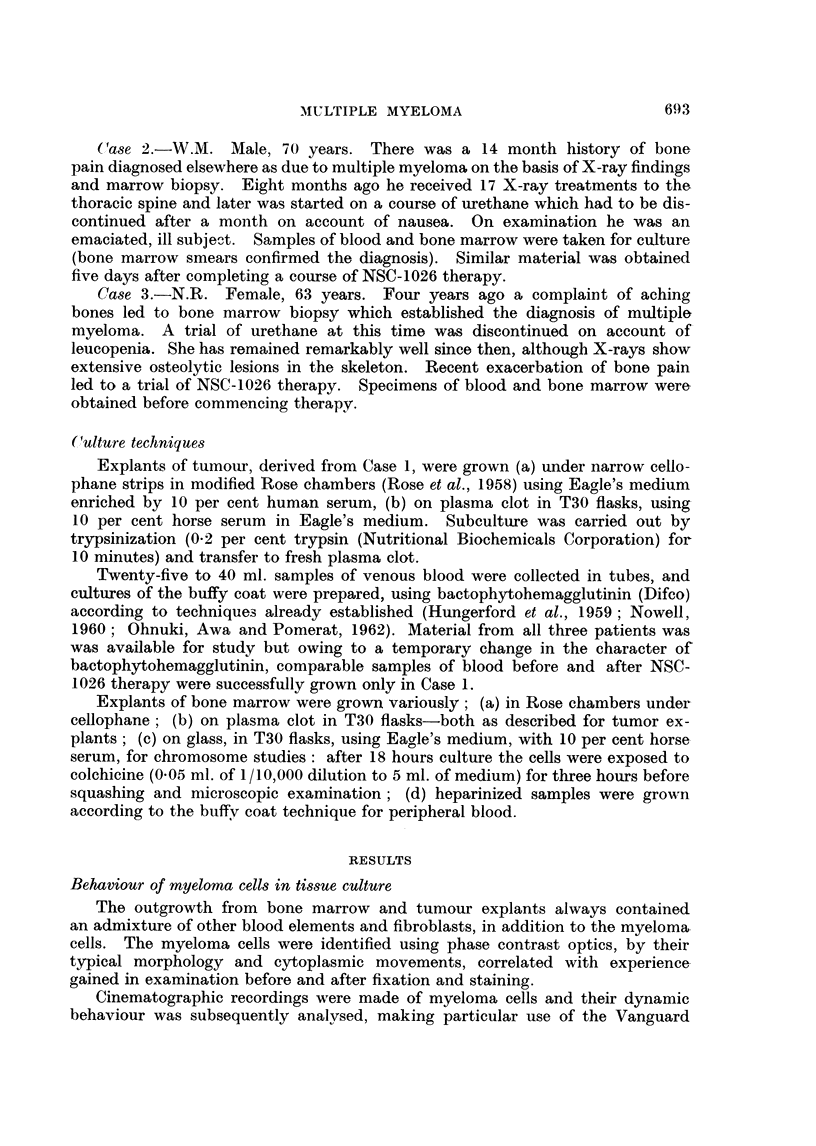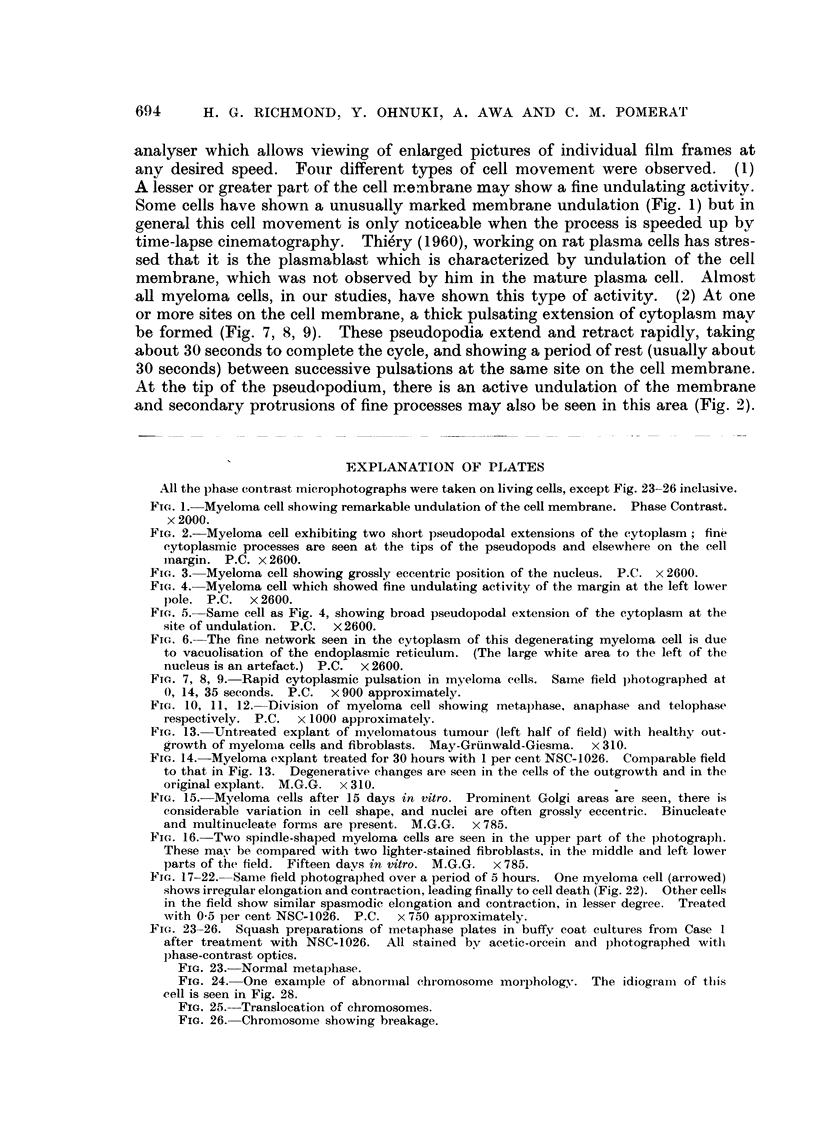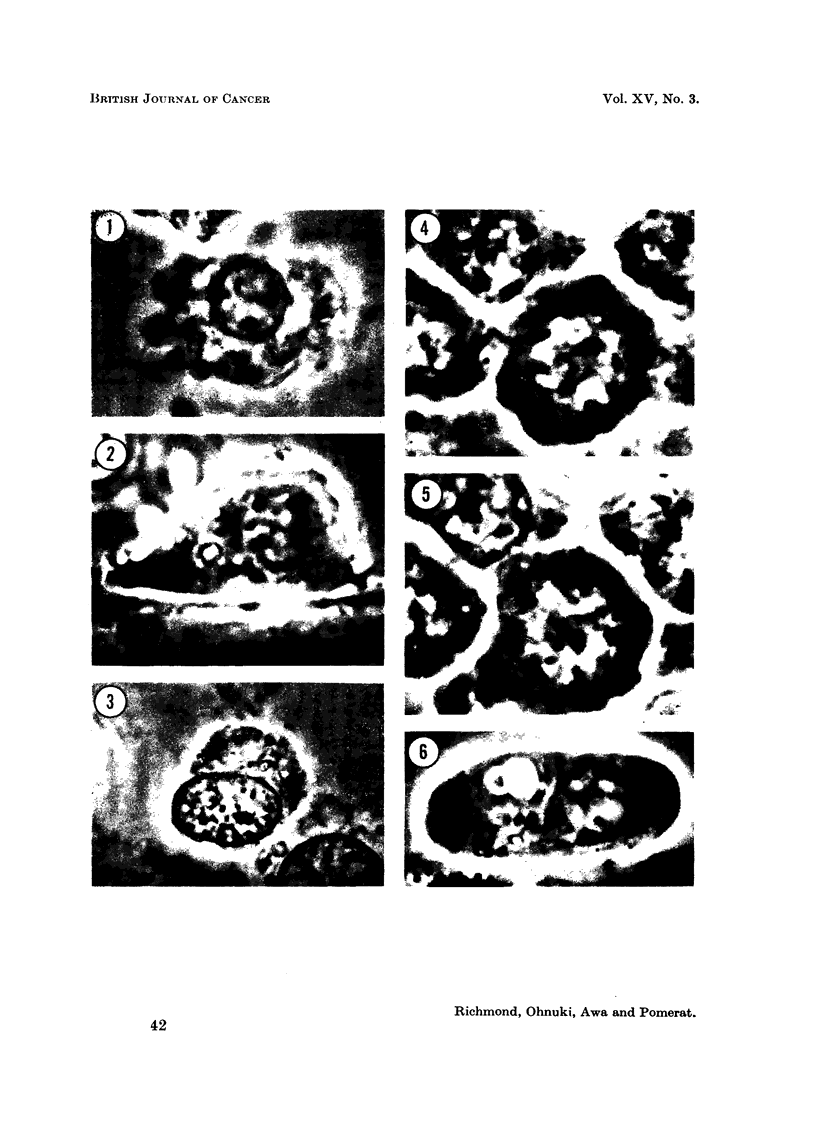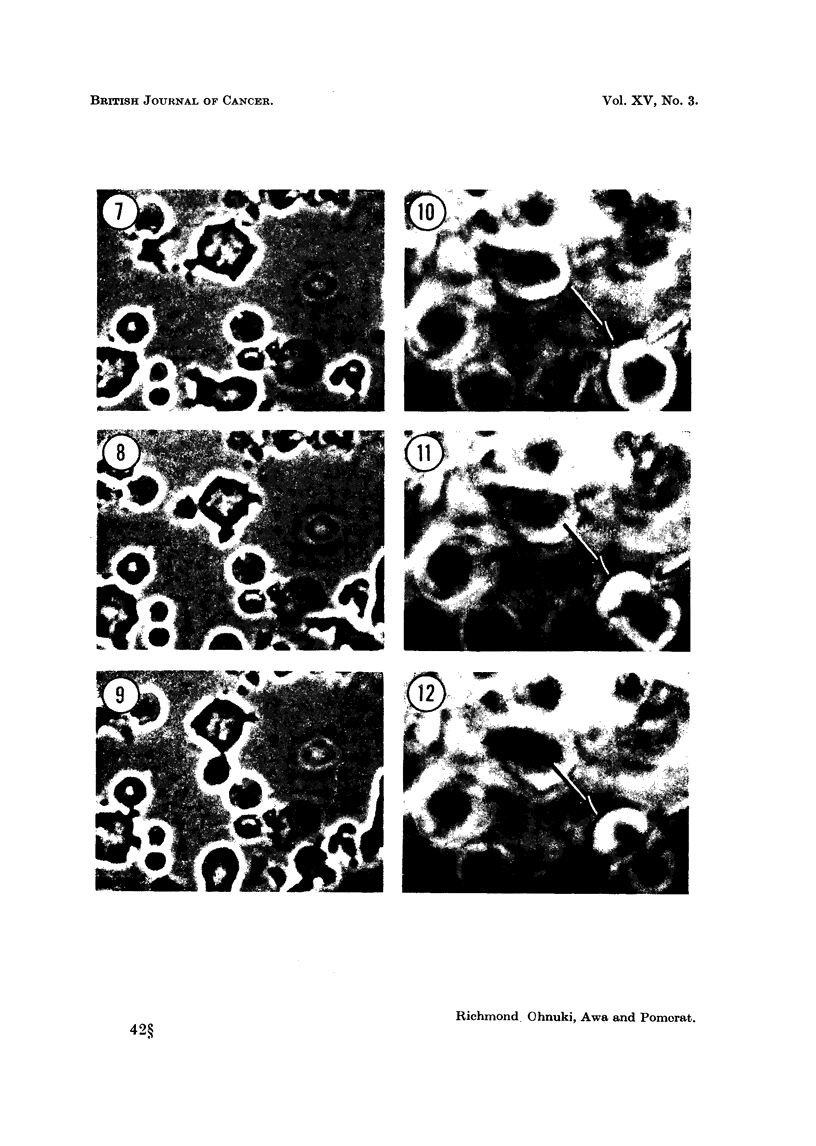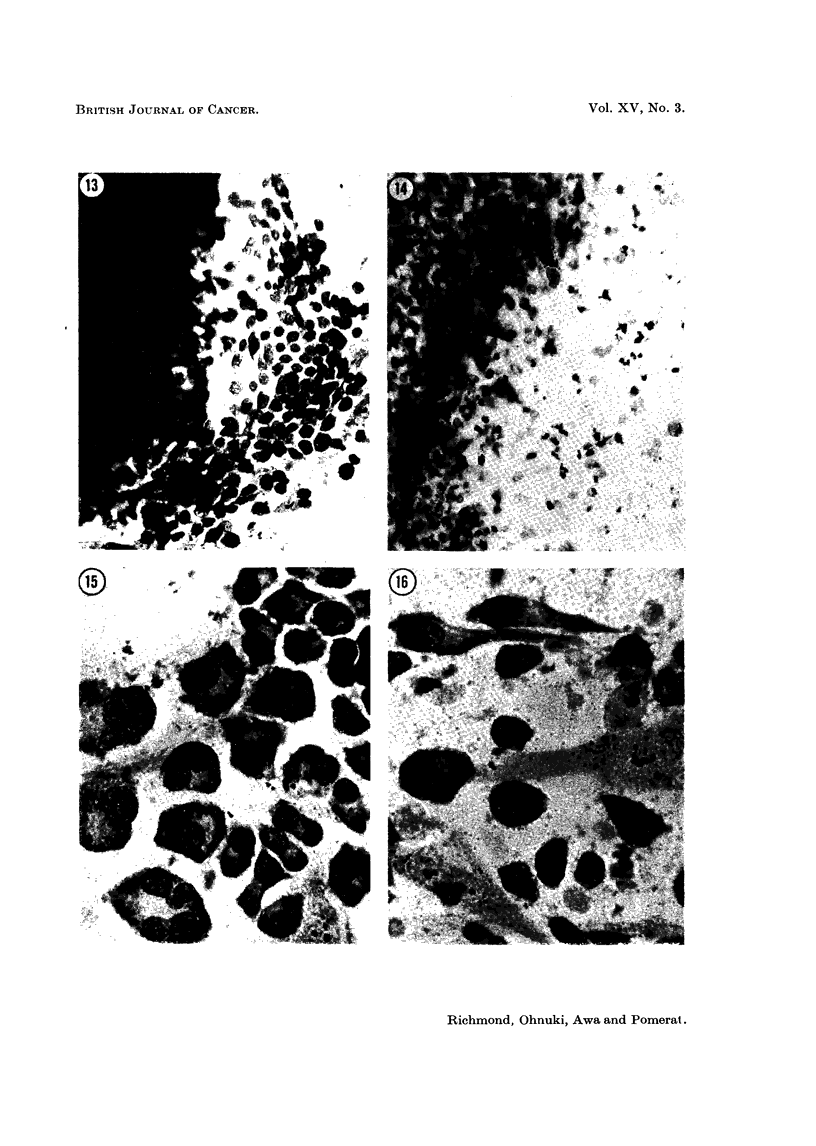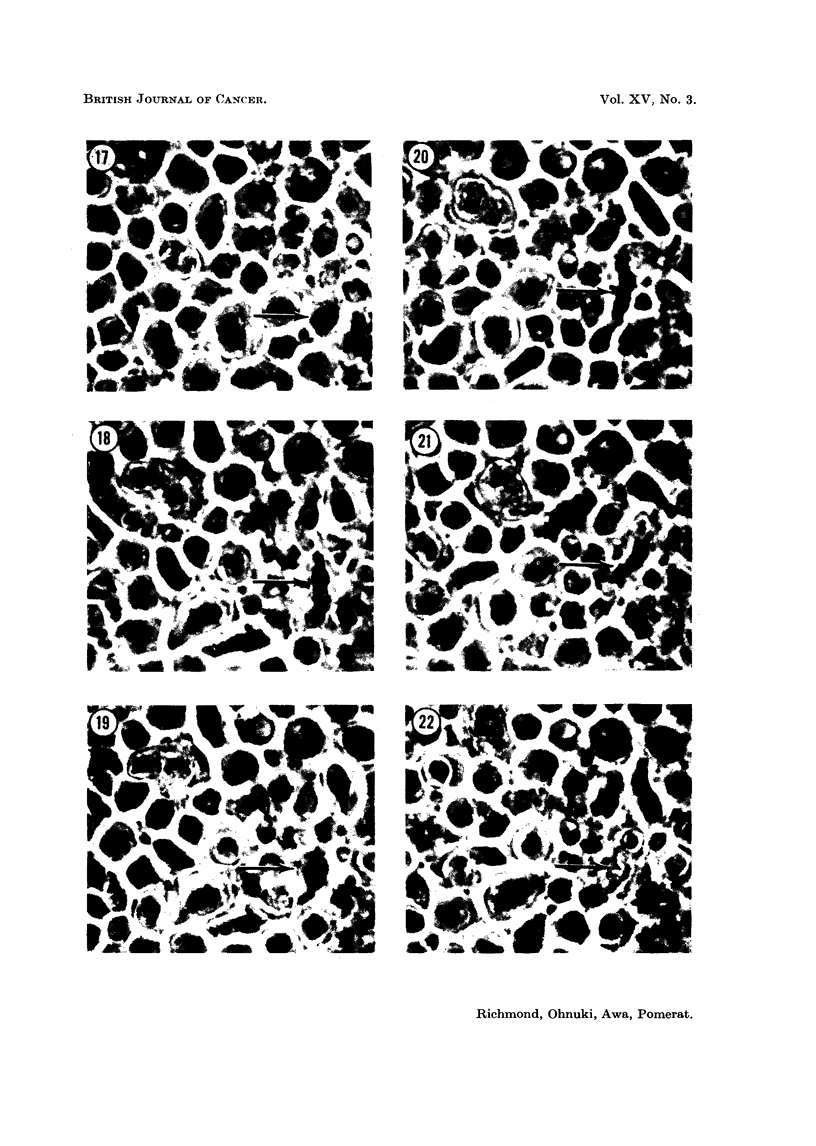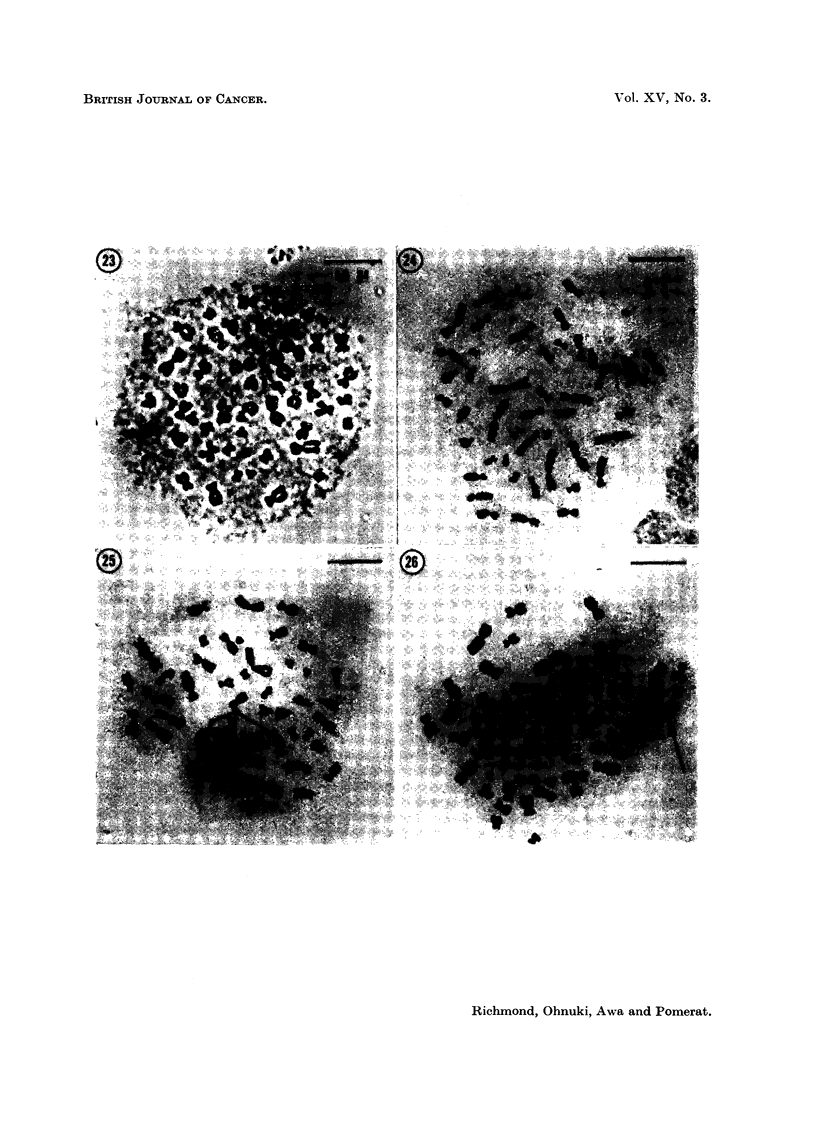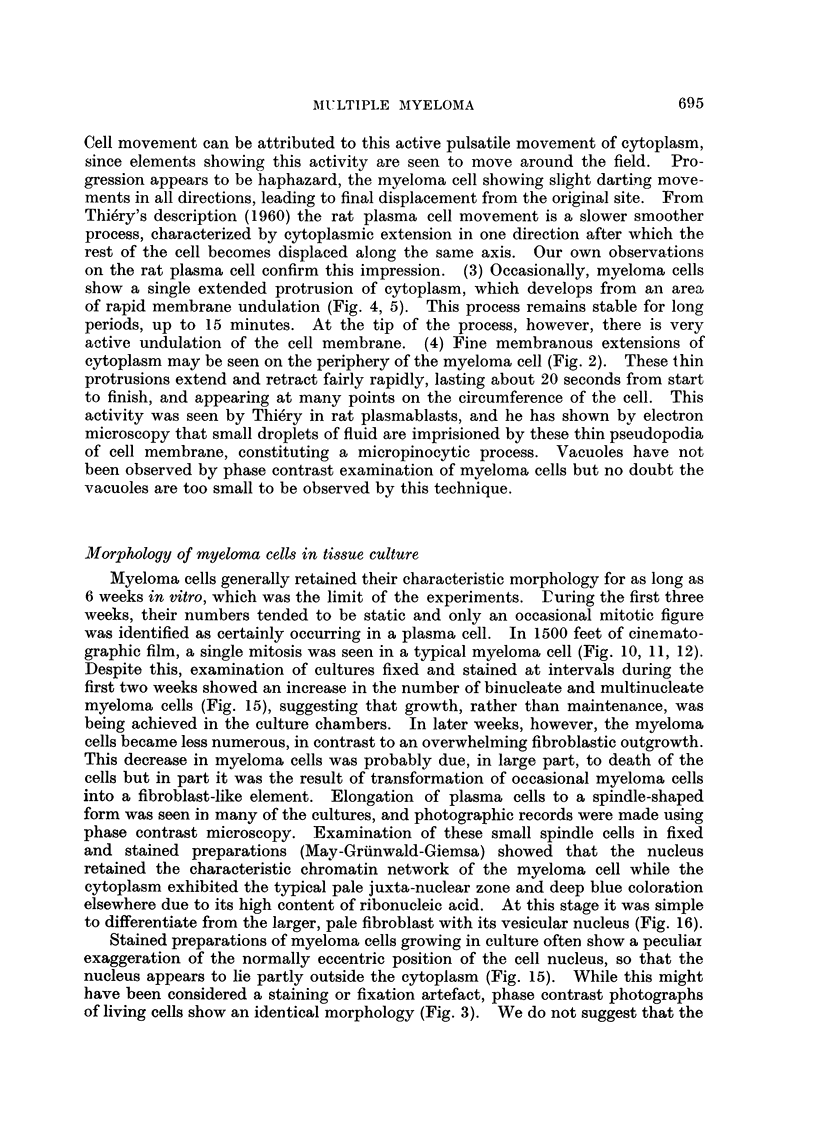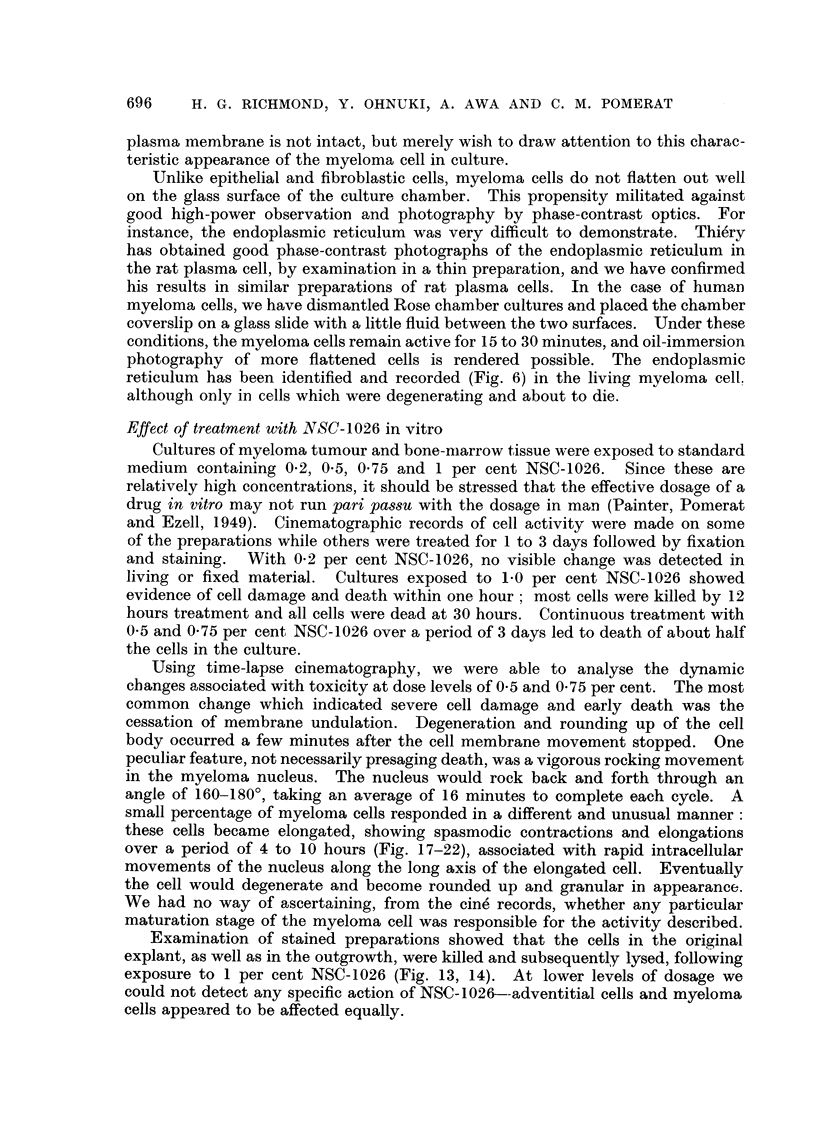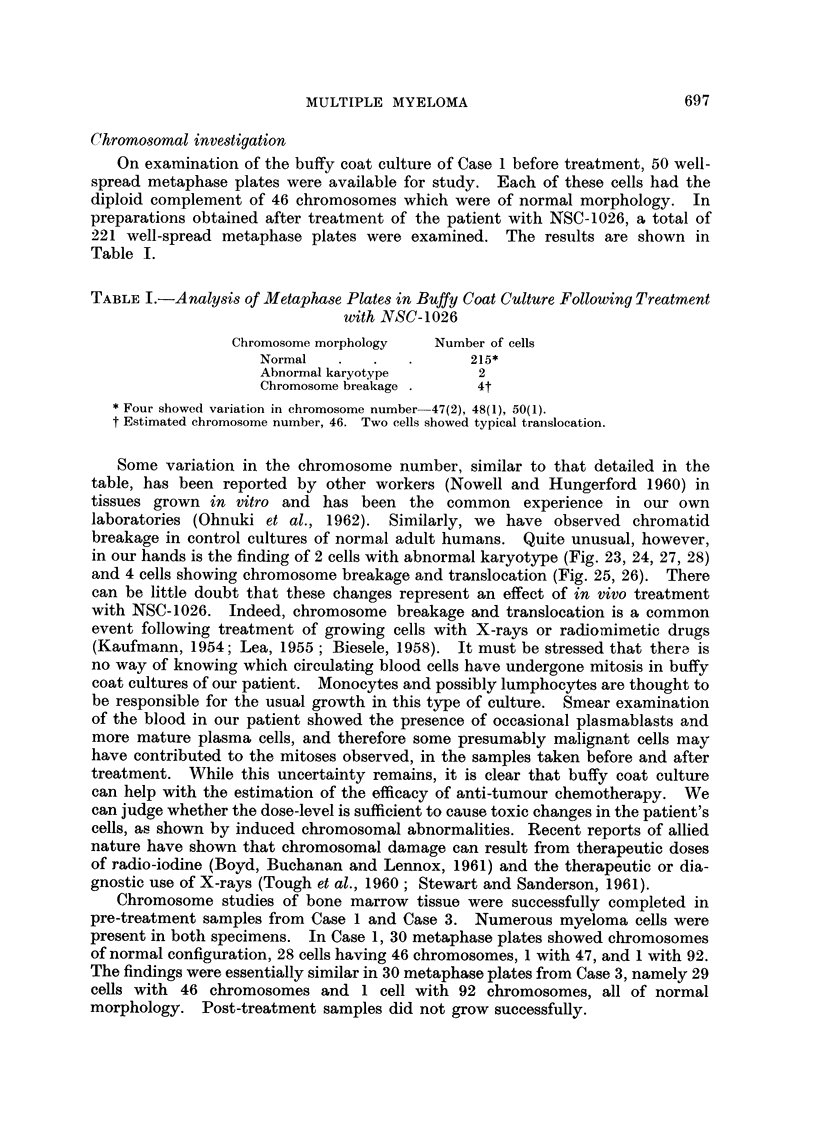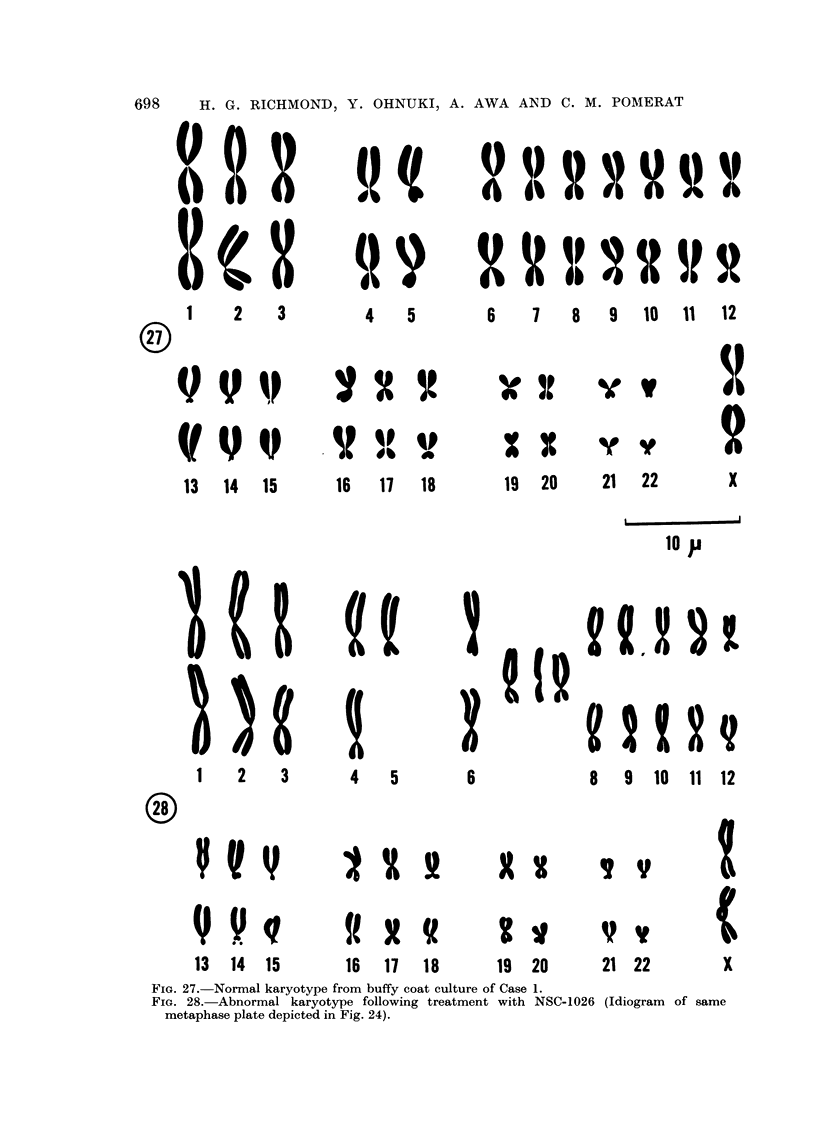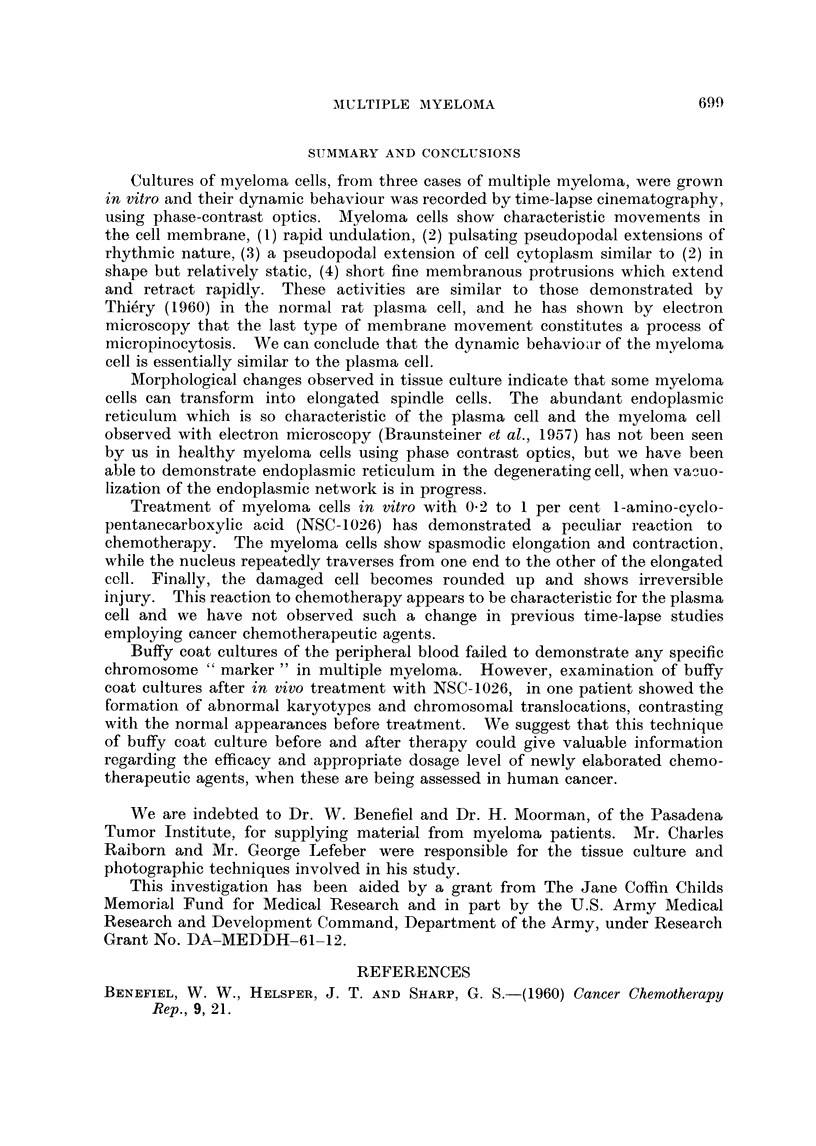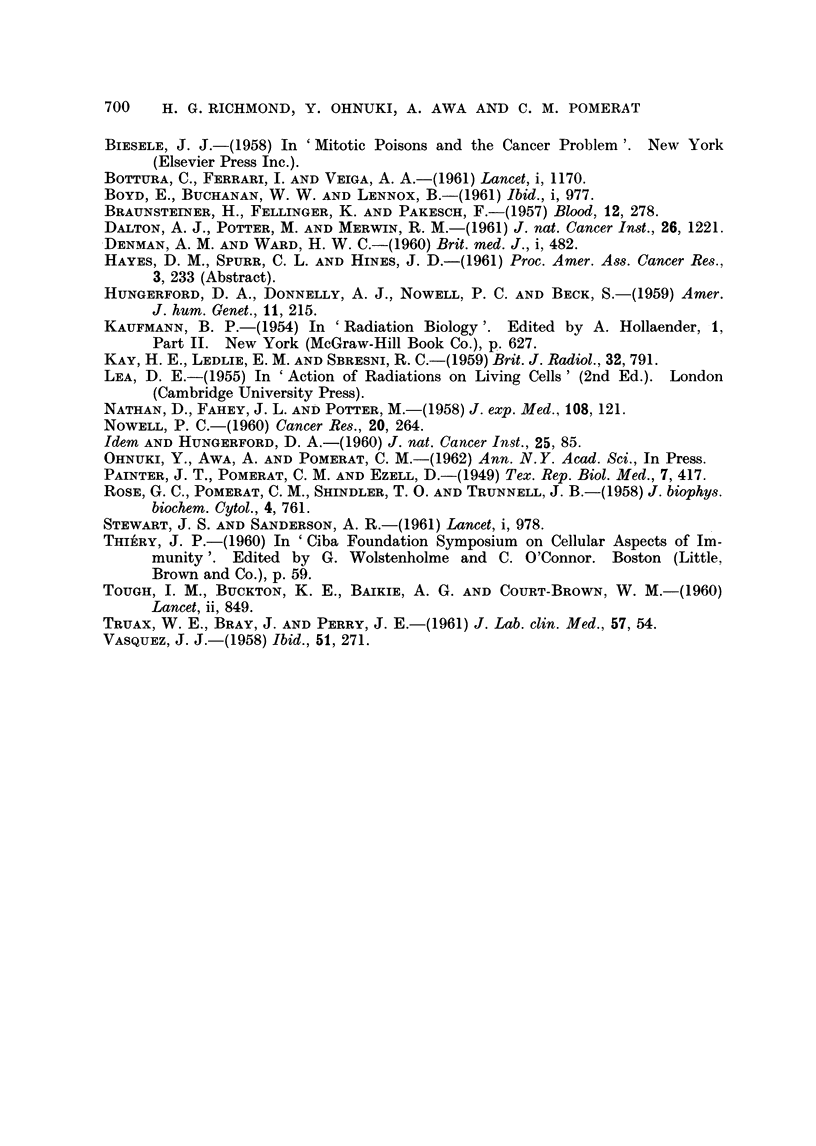# Multiple Myeloma—An In Vitro Study

**DOI:** 10.1038/bjc.1961.79

**Published:** 1961-09

**Authors:** H. G. Richmond, Y. Ohnuki, A. Awa, C. M. Pomerat

## Abstract

**Images:**


### MULTIPLE MYELOMA—AN *IN VITRO* STUDY

H. G. RICHMOND,* Y. OHNUKI,† A. AWA† AND C. M. POMERAT

*Pasadena Foundation for Medical Research, Pasadena, California, U.S.A.*

Received for publication July 19, 1961

RECENT experimental investigations of plasma cell myeloma have been directed mainly towards the elucidation of problems of protein synthesis in the myeloma cell (Nathan, Fahey and Potter, 1958 ; Vasquez, 1958 ; Truax, Bray and Perry 1961), and the effects of chemotherapy on tumour progression *in vivo* (Kay, Ledlie and Sbresni, 1959 ; Denman and Ward, 1960 ; Benefiel, Helsper and Sharp, 1960 ; Hayes, Spurr and Hines, 1961). Morphological studies have been concerned with details of ultrastructure as revealed by the electron microscope (Braunsteiner, Fellinger and Pakesch, 1957 ; Dalton, Potter and Merwin, 1961). In our present work we have investigated the dynamic morphology of myeloma cells, using phase contrast optics and time-lapse cinematography. We have been able to correlate these findings with similar work on the rat plasma cell carried out by Thiéry (1960) and personal observations on normal rat plasma cells.

The myeloma cells have been exposed *in vitro* to a recently developed chemotherapeutic compound, 1-aminocyclopentanecarboxylic acid (NSC-1026), which has proved useful in the treatment of multiple myelomatosis (Benefiel *et al.*, 1960) at a total dose level of 400–800 mg./kg. body weight. At the same time, preliminary work has been carried out on the chromosomal pattern of growing peripheral blood cells of myeloma patients. While no " marker " chromosomes were detected in our material, it is interesting to note that chromosomal aberrations have recently been established in sternal marrow preparations from a related disease, Waldenström's macroglobulinaemia (Bottura, Ferrari and Veiga, 1961). Samples of growing peripheral blood cells were also investigated before and after a course of NSC-1026.

### MATERIALS AND TECHNIQUES

Material for examination was obtained from three cases of multiple myeloma.

*Case* 1.—M.L. Female, 55 years. She complained of a lump ($5 \times 3 \times 3$ cm.) on the right side of the head. The tumour had been growing over a period of five months. X-ray demonstrated a destructive process in the underlying bone and histological examination of a biopsy showed the typical appearance of myeloma. Specimens of the tumour, bone marrow and peripheral blood were taken for study *in vitro*. The next day, the patient received the first of 10 daily intravenous injections of NSC-1026. Two days after the last injection repeat samples of blood, bone marrow and tumour were obtained.

* Fellow of The Jane Coffin Childs Memorial Fund for Medical Research. Permanent address: Department of Pathology, Royal Northern Infirmary, Inverness, Scotland.

† Fellows of the Tobacco Industry Research Committee. Permanent address : Zoological Institute, Hokkaido Univerity, Sapporo, Japan.

*Case* 2.—W.M. Male, 70 years. There was a 14 month history of bone pain diagnosed elsewhere as due to multiple myeloma on the basis of X-ray findings and marrow biopsy. Eight months ago he received 17 X-ray treatments to the thoracic spine and later was started on a course of urethane which had to be discontinued after a month on account of nausea. On examination he was an emaciated, ill subject. Samples of blood and bone marrow were taken for culture (bone marrow smears confirmed the diagnosis). Similar material was obtained five days after completing a course of NSC-1026 therapy.

*Case* 3.—N.R. Female, 63 years. Four years ago a complaint of aching bones led to bone marrow biopsy which established the diagnosis of multiple myeloma. A trial of urethane at this time was discontinued on account of leucopenia. She has remained remarkably well since then, although X-rays show extensive osteolytic lesions in the skeleton. Recent exacerbation of bone pain led to a trial of NSC-1026 therapy. Specimens of blood and bone marrow were obtained before commencing therapy.

*Culture techniques*

Explants of tumour, derived from Case 1, were grown (a) under narrow cellophane strips in modified Rose chambers (Rose *et al.*, 1958) using Eagle's medium enriched by 10 per cent human serum, (b) on plasma clot in T30 flasks, using 10 per cent horse serum in Eagle's medium. Subculture was carried out by trypsinization (0·2 per cent trypsin (Nutritional Biochemicals Corporation) for 10 minutes) and transfer to fresh plasma clot.

Twenty-five to 40 ml. samples of venous blood were collected in tubes, and cultures of the buffy coat were prepared, using bactophytohemagglutinin (Difco) according to techniques already established (Hungerford *et al.*, 1959 ; Nowell, 1960 ; Ohnuki, Awa and Pomerat, 1962). Material from all three patients was was available for study but owing to a temporary change in the character of bactophytohemagglutinin, comparable samples of blood before and after NSC-1026 therapy were successfully grown only in Case 1.

Explants of bone marrow were grown variously ; (a) in Rose chambers under cellophane ; (b) on plasma clot in T30 flasks—both as described for tumor explants ; (c) on glass, in T30 flasks, using Eagle's medium, with 10 per cent horse serum, for chromosome studies : after 18 hours culture the cells were exposed to colchicine (0·05 ml. of 1/10,000 dilution to 5 ml. of medium) for three hours before squashing and microscopic examination ; (d) heparinized samples were grown according to the buffy coat technique for peripheral blood.

### RESULTS

*Behaviour of myeloma cells in tissue culture*

The outgrowth from bone marrow and tumour explants always contained an admixture of other blood elements and fibroblasts, in addition to the myeloma cells. The myeloma cells were identified using phase contrast optics, by their typical morphology and cytoplasmic movements, correlated with experience gained in examination before and after fixation and staining.

Cinematographic recordings were made of myeloma cells and their dynamic behaviour was subsequently analysed, making particular use of the Vanguard analyser which allows viewing of enlarged pictures of individual film frames at any desired speed. Four different types of cell movement were observed. (1) A lesser or greater part of the cell membrane may show a fine undulating activity. Some cells have shown a unusually marked membrane undulation (Fig. 1) but in general this cell movement is only noticeable when the process is speeded up by time-lapse cinematography. Thiéry (1960), working on rat plasma cells has stressed that it is the plasmablast which is characterized by undulation of the cell membrane, which was not observed by him in the mature plasma cell. Almost all myeloma cells, in our studies, have shown this type of activity. (2) At one or more sites on the cell membrane, a thick pulsating extension of cytoplasm may be formed (Fig. 7, 8, 9). These pseudopodia extend and retract rapidly, taking about 30 seconds to complete the cycle, and showing a period of rest (usually about 30 seconds) between successive pulsations at the same site on the cell membrane. At the tip of the pseudopodium, there is an active undulation of the membrane and secondary protrusions of fine processes may also be seen in this area (Fig. 2).

---

### EXPLANATION OF PLATES

All the phase contrast microphotographs were taken on living cells, except Fig. 23–26 inclusive.

FIG. 1.—Myeloma cell showing remarkable undulation of the cell membrane. Phase Contrast. ×2000.

FIG. 2.—Myeloma cell exhibiting two short pseudopodal extensions of the cytoplasm ; fine cytoplasmic processes are seen at the tips of the pseudopods and elsewhere on the cell margin. P.C. ×2600.

FIG. 3.—Myeloma cell showing grossly eccentric position of the nucleus. P.C. ×2600.

FIG. 4.—Myeloma cell which showed fine undulating activity of the margin at the left lower pole. P.C. ×2600.

FIG. 5.—Same cell as Fig. 4, showing broad pseudopodal extension of the cytoplasm at the site of undulation. P.C. ×2600.

FIG. 6.—The fine network seen in the cytoplasm of this degenerating myeloma cell is due to vacuolisation of the endoplasmic reticulum. (The large white area to the left of the nucleus is an artefact.) P.C. ×2600.

FIG. 7, 8, 9.—Rapid cytoplasmic pulsation in myeloma cells. Same field photographed at 0, 14, 35 seconds. P.C. ×900 approximately.

FIG. 10, 11, 12.—Division of myeloma cell showing metaphase, anaphase and telophase respectively. P.C. ×1000 approximately.

FIG. 13.—Untreated explant of myelomatous tumour (left half of field) with healthy outgrowth of myeloma cells and fibroblasts. May-Grünwald-Giesma. ×310.

FIG. 14.—Myeloma explant treated for 30 hours with 1 per cent NSC-1026. Comparable field to that in Fig. 13. Degenerative changes are seen in the cells of the outgrowth and in the original explant. M.G.G. ×310.

FIG. 15.—Myeloma cells after 15 days *in vitro*. Prominent Golgi areas are seen, there is considerable variation in cell shape, and nuclei are often grossly eccentric. Binucleate and multinucleate forms are present. M.G.G. ×785.

FIG. 16.—Two spindle-shaped myeloma cells are seen in the upper part of the photograph. These may be compared with two lighter-stained fibroblasts, in the middle and left lower parts of the field. Fifteen days *in vitro*. M.G.G. ×785.

FIG. 17–22.—Same field photographed over a period of 5 hours. One myeloma cell (arrowed) shows irregular elongation and contraction, leading finally to cell death (Fig. 22). Other cells in the field show similar spasmodic elongation and contraction, in lesser degree. Treated with 0·5 per cent NSC-1026. P.C. ×750 approximately.

FIG. 23–26. Squash preparations of metaphase plates in buffy coat cultures from Case 1 after treatment with NSC-1026. All stained by acetic-orcein and photographed with phase-contrast optics.

FIG. 23.—Normal metaphase.

FIG. 24.—One example of abnormal chromosome morphology. The idiogram of this cell is seen in Fig. 28.

FIG. 25.—Translocation of chromosomes.

FIG. 26.—Chromosome showing breakage.

1
2
3
4
5
6

7

8

9

10

11

12

Richmond, Ohnuki, Awa and Pomerat.

Richmond, Ohnuki, Awa, Pomerat.

Richmond, Ohnuki, Awa and Pomerat.

Cell movement can be attributed to this active pulsatile movement of cytoplasm, since elements showing this activity are seen to move around the field. Progression appears to be haphazard, the myeloma cell showing slight darting movements in all directions, leading to final displacement from the original site. From Thiéry's description (1960) the rat plasma cell movement is a slower smoother process, characterized by cytoplasmic extension in one direction after which the rest of the cell becomes displaced along the same axis. Our own observations on the rat plasma cell confirm this impression. (3) Occasionally, myeloma cells show a single extended protrusion of cytoplasm, which develops from an area of rapid membrane undulation (Fig. 4, 5). This process remains stable for long periods, up to 15 minutes. At the tip of the process, however, there is very active undulation of the cell membrane. (4) Fine membranous extensions of cytoplasm may be seen on the periphery of the myeloma cell (Fig. 2). These thin protrusions extend and retract fairly rapidly, lasting about 20 seconds from start to finish, and appearing at many points on the circumference of the cell. This activity was seen by Thiéry in rat plasmablasts, and he has shown by electron microscopy that small droplets of fluid are imprisioned by these thin pseudopodia of cell membrane, constituting a micropinocytic process. Vacuoles have not been observed by phase contrast examination of myeloma cells but no doubt the vacuoles are too small to be observed by this technique.

*Morphology of myeloma cells in tissue culture*

Myeloma cells generally retained their characteristic morphology for as long as 6 weeks *in vitro*, which was the limit of the experiments. During the first three weeks, their numbers tended to be static and only an occasional mitotic figure was identified as certainly occurring in a plasma cell. In 1500 feet of cinematographic film, a single mitosis was seen in a typical myeloma cell (Fig. 10, 11, 12). Despite this, examination of cultures fixed and stained at intervals during the first two weeks showed an increase in the number of binucleate and multinucleate myeloma cells (Fig. 15), suggesting that growth, rather than maintenance, was being achieved in the culture chambers. In later weeks, however, the myeloma cells became less numerous, in contrast to an overwhelming fibroblastic outgrowth. This decrease in myeloma cells was probably due, in large part, to death of the cells but in part it was the result of transformation of occasional myeloma cells into a fibroblast-like element. Elongation of plasma cells to a spindle-shaped form was seen in many of the cultures, and photographic records were made using phase contrast microscopy. Examination of these small spindle cells in fixed and stained preparations (May-Grünwald-Giemsa) showed that the nucleus retained the characteristic chromatin network of the myeloma cell while the cytoplasm exhibited the typical pale juxta-nuclear zone and deep blue coloration elsewhere due to its high content of ribonucleic acid. At this stage it was simple to differentiate from the larger, pale fibroblast with its vesicular nucleus (Fig. 16).

Stained preparations of myeloma cells growing in culture often show a peculiar exaggeration of the normally eccentric position of the cell nucleus, so that the nucleus appears to lie partly outside the cytoplasm (Fig. 15). While this might have been considered a staining or fixation artefact, phase contrast photographs of living cells show an identical morphology (Fig. 3). We do not suggest that the plasma membrane is not intact, but merely wish to draw attention to this characteristic appearance of the myeloma cell in culture.

Unlike epithelial and fibroblastic cells, myeloma cells do not flatten out well on the glass surface of the culture chamber. This propensity militated against good high-power observation and photography by phase-contrast optics. For instance, the endoplasmic reticulum was very difficult to demonstrate. Thiéry has obtained good phase-contrast photographs of the endoplasmic reticulum in the rat plasma cell, by examination in a thin preparation, and we have confirmed his results in similar preparations of rat plasma cells. In the case of human myeloma cells, we have dismantled Rose chamber cultures and placed the chamber coverslip on a glass slide with a little fluid between the two surfaces. Under these conditions, the myeloma cells remain active for 15 to 30 minutes, and oil-immersion photography of more flattened cells is rendered possible. The endoplasmic reticulum has been identified and recorded (Fig. 6) in the living myeloma cell, although only in cells which were degenerating and about to die.

*Effect of treatment with NSC*-1026 in vitro

Cultures of myeloma tumour and bone-marrow tissue were exposed to standard medium containing 0·2, 0·5, 0·75 and 1 per cent NSC-1026. Since these are relatively high concentrations, it should be stressed that the effective dosage of a drug *in vitro* may not run *pari passu* with the dosage in man (Painter, Pomerat and Ezell, 1949). Cinematographic records of cell activity were made on some of the preparations while others were treated for 1 to 3 days followed by fixation and staining. With 0·2 per cent NSC-1026, no visible change was detected in living or fixed material. Cultures exposed to 1·0 per cent NSC-1026 showed evidence of cell damage and death within one hour ; most cells were killed by 12 hours treatment and all cells were dead at 30 hours. Continuous treatment with 0·5 and 0·75 per cent NSC-1026 over a period of 3 days led to death of about half the cells in the culture.

Using time-lapse cinematography, we were able to analyse the dynamic changes associated with toxicity at dose levels of 0·5 and 0·75 per cent. The most common change which indicated severe cell damage and early death was the cessation of membrane undulation. Degeneration and rounding up of the cell body occurred a few minutes after the cell membrane movement stopped. One peculiar feature, not necessarily presaging death, was a vigorous rocking movement in the myeloma nucleus. The nucleus would rock back and forth through an angle of 160–180°, taking an average of 16 minutes to complete each cycle. A small percentage of myeloma cells responded in a different and unusual manner : these cells became elongated, showing spasmodic contractions and elongations over a period of 4 to 10 hours (Fig. 17–22), associated with rapid intracellular movements of the nucleus along the long axis of the elongated cell. Eventually the cell would degenerate and become rounded up and granular in appearance. We had no way of ascertaining, from the ciné records, whether any particular maturation stage of the myeloma cell was responsible for the activity described.

Examination of stained preparations showed that the cells in the original explant, as well as in the outgrowth, were killed and subsequently lysed, following exposure to 1 per cent NSC-1026 (Fig. 13, 14). At lower levels of dosage we could not detect any specific action of NSC-1026—adventitial cells and myeloma cells appeared to be affected equally.

*Chromosomal investigation*

On examination of the buffy coat culture of Case 1 before treatment, 50 well-spread metaphase plates were available for study. Each of these cells had the diploid complement of 46 chromosomes which were of normal morphology. In preparations obtained after treatment of the patient with NSC-1026, a total of 221 well-spread metaphase plates were examined. The results are shown in Table I.

TABLE I.—*Analysis of Metaphase Plates in Buffy Coat Culture Following Treatment with NSC-1026*

| Chromosome morphology | Number of cells |
|---|---|
| Normal . . . | 215* |
| Abnormal karyotype | 2 |
| Chromosome breakage . | 4† |

* Four showed variation in chromosome number—47(2), 48(1), 50(1).
† Estimated chromosome number, 46. Two cells showed typical translocation.

Some variation in the chromosome number, similar to that detailed in the table, has been reported by other workers (Nowell and Hungerford 1960) in tissues grown *in vitro* and has been the common experience in our own laboratories (Ohnuki *et al.*, 1962). Similarly, we have observed chromatid breakage in control cultures of normal adult humans. Quite unusual, however, in our hands is the finding of 2 cells with abnormal karyotype (Fig. 23, 24, 27, 28) and 4 cells showing chromosome breakage and translocation (Fig. 25, 26). There can be little doubt that these changes represent an effect of *in vivo* treatment with NSC-1026. Indeed, chromosome breakage and translocation is a common event following treatment of growing cells with X-rays or radiomimetic drugs (Kaufmann, 1954; Lea, 1955 ; Biesele, 1958). It must be stressed that there is no way of knowing which circulating blood cells have undergone mitosis in buffy coat cultures of our patient. Monocytes and possibly lumphocytes are thought to be responsible for the usual growth in this type of culture. Smear examination of the blood in our patient showed the presence of occasional plasmablasts and more mature plasma cells, and therefore some presumably malignant cells may have contributed to the mitoses observed, in the samples taken before and after treatment. While this uncertainty remains, it is clear that buffy coat culture can help with the estimation of the efficacy of anti-tumour chemotherapy. We can judge whether the dose-level is sufficient to cause toxic changes in the patient's cells, as shown by induced chromosomal abnormalities. Recent reports of allied nature have shown that chromosomal damage can result from therapeutic doses of radio-iodine (Boyd, Buchanan and Lennox, 1961) and the therapeutic or diagnostic use of X-rays (Tough *et al.*, 1960 ; Stewart and Sanderson, 1961).

Chromosome studies of bone marrow tissue were successfully completed in pre-treatment samples from Case 1 and Case 3. Numerous myeloma cells were present in both specimens. In Case 1, 30 metaphase plates showed chromosomes of normal configuration, 28 cells having 46 chromosomes, 1 with 47, and 1 with 92. The findings were essentially similar in 30 metaphase plates from Case 3, namely 29 cells with 46 chromosomes and 1 cell with 92 chromosomes, all of normal morphology. Post-treatment samples did not grow successfully.

FIG. 27.—Normal karyotype from buffy coat culture of Case 1.
FIG. 28.—Abnormal karyotype following treatment with NSC-1026 (Idiogram of same metaphase plate depicted in Fig. 24).

### SUMMARY AND CONCLUSIONS

Cultures of myeloma cells, from three cases of multiple myeloma, were grown *in vitro* and their dynamic behaviour was recorded by time-lapse cinematography, using phase-contrast optics. Myeloma cells show characteristic movements in the cell membrane, (1) rapid undulation, (2) pulsating pseudopodal extensions of rhythmic nature, (3) a pseudopodal extension of cell cytoplasm similar to (2) in shape but relatively static, (4) short fine membranous protrusions which extend and retract rapidly. These activities are similar to those demonstrated by Thiéry (1960) in the normal rat plasma cell, and he has shown by electron microscopy that the last type of membrane movement constitutes a process of micropinocytosis. We can conclude that the dynamic behaviour of the myeloma cell is essentially similar to the plasma cell.

Morphological changes observed in tissue culture indicate that some myeloma cells can transform into elongated spindle cells. The abundant endoplasmic reticulum which is so characteristic of the plasma cell and the myeloma cell observed with electron microscopy (Braunsteiner *et al.*, 1957) has not been seen by us in healthy myeloma cells using phase contrast optics, but we have been able to demonstrate endoplasmic reticulum in the degenerating cell, when vacuolization of the endoplasmic network is in progress.

Treatment of myeloma cells *in vitro* with 0·2 to 1 per cent 1-amino-cyclopentanecarboxylic acid (NSC-1026) has demonstrated a peculiar reaction to chemotherapy. The myeloma cells show spasmodic elongation and contraction, while the nucleus repeatedly traverses from one end to the other of the elongated cell. Finally, the damaged cell becomes rounded up and shows irreversible injury. This reaction to chemotherapy appears to be characteristic for the plasma cell and we have not observed such a change in previous time-lapse studies employing cancer chemotherapeutic agents.

Buffy coat cultures of the peripheral blood failed to demonstrate any specific chromosome " marker " in multiple myeloma. However, examination of buffy coat cultures after *in vivo* treatment with NSC-1026, in one patient showed the formation of abnormal karyotypes and chromosomal translocations, contrasting with the normal appearances before treatment. We suggest that this technique of buffy coat culture before and after therapy could give valuable information regarding the efficacy and appropriate dosage level of newly elaborated chemotherapeutic agents, when these are being assessed in human cancer.

We are indebted to Dr. W. Benefiel and Dr. H. Moorman, of the Pasadena Tumor Institute, for supplying material from myeloma patients. Mr. Charles Raiborn and Mr. George Lefeber were responsible for the tissue culture and photographic techniques involved in his study.

This investigation has been aided by a grant from The Jane Coffin Childs Memorial Fund for Medical Research and in part by the U.S. Army Medical Research and Development Command, Department of the Army, under Research Grant No. DA–MEDDH–61–12.

### REFERENCES

BENEFIEL, W. W., HELSPER, J. T. AND SHARP, G. S.—(1960) *Cancer Chemotherapy Rep.*, **9**, 21.

BIESELE, J. J.—(1958) In 'Mitotic Poisons and the Cancer Problem'. New York (Elsevier Press Inc.).
BOTTURA, C., FERRARI, I. AND VEIGA, A. A.—(1961) *Lancet*, i, 1170.
BOYD, E., BUCHANAN, W. W. AND LENNOX, B.—(1961) *Ibid.*, i, 977.
BRAUNSTEINER, H., FELLINGER, K. AND PAKESCH, F.—(1957) *Blood*, **12**, 278.
DALTON, A. J., POTTER, M. AND MERWIN, R. M.—(1961) *J. nat. Cancer Inst.*, **26**, 1221.
DENMAN, A. M. AND WARD, H. W. C.—(1960) *Brit. med. J.*, i, 482.
HAYES, D. M., SPURR, C. L. AND HINES, J. D.—(1961) *Proc. Amer. Ass. Cancer Res.*, **3**, 233 (Abstract).
HUNGERFORD, D. A., DONNELLY, A. J., NOWELL, P. C. AND BECK, S.—(1959) *Amer. J. hum. Genet.*, **11**, 215.
KAUFMANN, B. P.—(1954) In 'Radiation Biology'. Edited by A. Hollaender, **1**, Part II. New York (McGraw-Hill Book Co.), p. 627.
KAY, H. E., LEDLIE, E. M. AND SBRESNI, R. C.—(1959) *Brit. J. Radiol.*, **32**, 791.
LEA, D. E.—(1955) In 'Action of Radiations on Living Cells' (2nd Ed.). London (Cambridge University Press).
NATHAN, D., FAHEY, J. L. AND POTTER, M.—(1958) *J. exp. Med.*, **108**, 121.
NOWELL, P. C.—(1960) *Cancer Res.*, **20**, 264.
*Idem* AND HUNGERFORD, D. A.—(1960) *J. nat. Cancer Inst.*, **25**, 85.
OHNUKI, Y., AWA, A. AND POMERAT, C. M.—(1962) *Ann. N.Y. Acad. Sci.*, In Press.
PAINTER, J. T., POMERAT, C. M. AND EZELL, D.—(1949) *Tex. Rep. Biol. Med.*, **7**, 417.
ROSE, G. C., POMERAT, C. M., SHINDLER, T. O. AND TRUNNELL, J. B.—(1958) *J. biophys. biochem. Cytol.*, **4**, 761.
STEWART, J. S. AND SANDERSON, A. R.—(1961) *Lancet*, i, 978.
THIÉRY, J. P.—(1960) In 'Ciba Foundation Symposium on Cellular Aspects of Immunity'. Edited by G. Wolstenholme and C. O'Connor. Boston (Little, Brown and Co.), p. 59.
TOUGH, I. M., BUCKTON, K. E., BAIKIE, A. G. AND COURT-BROWN, W. M.—(1960) *Lancet*, ii, 849.
TRUAX, W. E., BRAY, J. AND PERRY, J. E.—(1961) *J. Lab. clin. Med.*, **57**, 54.
VASQUEZ, J. J.—(1958) *Ibid.*, **51**, 271.